# Investigation of the effect of anti-PIA/PNAG antibodies on biofilm formation in *Escherichia coli*

**DOI:** 10.3389/fmicb.2025.1552670

**Published:** 2025-03-06

**Authors:** Mina Shirmohammadpour, Mohammad Reza Mehrasbi, Nader Noshiranzadeh, Davoud Afshar, Kamyar Mansori, Bahman Mirzaei

**Affiliations:** ^1^Department of Microbiology and Virology, Faculty of Medicine, Zanjan University of Medical Sciences, Zanjan, Iran; ^2^Student Research Committee, Department of Medical Microbiology and Virology, School of Medicine, Zanjan University of Medical Sciences, Zanjan, Iran; ^3^Department of Environmental Health Engineering, School of Public Health, Zanjan University of Medical Sciences, Zanjan, Iran; ^4^Department of Chemistry, Faculty of Sciences, University of Zanjan, Zanjan, Iran; ^5^Department of Biostatistics and Epidemiology, School of Medicine, Zanjan University of Medical Sciences, Zanjan, Iran

**Keywords:** *Escherichia coli*, *Staphylococcus epidermidis*, polysaccharide vaccine, immunization, PIA, PNAG

## Abstract

Polysaccharide Intercellular Adhesin (PIA), a surface polysaccharide produced by *Staphylococcus aureus* and *Staphylococcus epidermidis*, is a compelling target for opsonic and protective antibodies against these bacteria. *Escherichia coli* has recently made an exopolysaccharide called poly-*β*(1,6)-*N*-acetylglucosamine (PNAG), biochemically indistinguishable from PIA. This study investigated the effect of antibodies generated against PNAG on biofilm formation and the opsonization activity of secreted antibodies in *Escherichia coli*. Following purification and structural confirmation of PIA polysaccharide from producing *Staphylococcus epidermidis*, the ability to inhibit biofilm and the function of secreted antibodies for the mentioned polysaccharide were evaluated using semi-quantitative methods in a mouse model. Subsequently, the opsonic activity of antibodies targeting *Escherichia coli* strain ATCC 25922 was evaluated. The extracted polysaccharide was confirmed using FTIR, NMR, and colorimetric methods, and the results showed that the purified PIA induced protective antibodies with 40.48% opsonization properties in *E. coli*. The sera of the PIA-immunized groups showed a significant increase in antibody production and protective IgG titer levels compared to the control group. Also, the antibodies produced showed a substantial difference in inhibiting biofilm production *in vitro* compared to non-immunized serum. Antibodies directed against PIA with a lethality of 40.48% showed a significant effect on the absence of biofilm formation in *E. coli*. Despite the opsonic properties of the antibodies for *E. coli*, the simultaneous impact of these antibodies on infections caused by *S. epidermidis* and *E. coli* may have a role that requires further investigation and studies in animal models.

## 1 Introduction

Biofilms are complex consortia of microorganisms that adhere to surfaces and are encased in a protective matrix composed of extracellular polymeric substances (EPS). This unique structure allows biofilms to rise in different environments, such as medical devices and human tissues, significantly impacting health outcomes. The significance of biofilms in medicine is underscored by their involvement in chronic infections and their association with heightened antibiotic resistance. For example, biofilms are thought to play a role in approximately 65–80% of bacterial infections, particularly those associated with medical implants such as catheters and prosthetic joints (Mendhe et al., 2023; Ali et al., 2023).

The biofilm EPS matrix protects bacteria from host immune responses and increases their resistance to antimicrobial agents, making infections difficult to treat. Biofilms can harbor persistent cells that enter an inactive state, which helps them prevail in antibiotic treatments without genetic changes. These cells can re-establish active infections after the end of treatment (Zhang et al., 2022). Furthermore, biofilms complicate diagnosis and treatment strategies due to their heterogeneous nature and ability to evade conventional therapeutic approaches (Mendhe et al., 2023; Ali et al., 2023).

Emerging research is concentrated on developing innovative strategies to combat biofilm-associated infections, including developing anti-biofilm agents and therapies that interrupt biofilm formation or enhance the capability of extant antibiotics (Ali et al., 2023; Zhang et al., 2022). Understanding the mechanisms behind biofilm development and persistence is critical to improving patient outcomes and addressing the challenges posed by these resilient microbial communities in clinical settings (Mendhe et al., 2023).

*Escherichia coli* (*E. coli*) is a versatile bacterium commonly found in the gastrointestinal tract of humans and animals, with some strains capable of forming biofilms, structured societies of bacteria tied to surfaces, and enclosed in a protective matrix. Biofilm formation in *E. coli* is mediated by a multitude of factors that enhance surface adhesion and promote community stability. Recent investigations have underscored the importance of biofilm-forming *E. coli*, especially in clinical settings. Research shows that biofilm-forming *E. coli* isolates show higher antibiotic resistance than non-biofilm-forming ones, which complicates treatment strategies for infections caused by these bacteria. Understanding the molecular procedures underlying biofilm formation in *E. coli* is crucial for the evolution of effective therapeutic interventions to combat biofilm-associated infections and reduce their impact on public health (Fang et al., 2018; Hassuna et al., 2024; Katongole et al., 2020).

Poly-*β*(1,6)-*N*-acetylglucosamine (PNAG) is an exopolysaccharide in *E. coli* that significantly contributes to biofilm formation. PNAG, synthesized through the *pgaABCD* operon, enhances the capability of bacteria to stick to surfaces and make structured communities, which are essential for biofilm development. Recent studies have shown that PNAG promotes cell-to-cell aggregation and stabilizes the biofilm matrix, thereby providing structural integrity and protection against environmental stresses and antimicrobial agents. The presence of PNAG significantly enhances the adhesion capabilities of biofilm-forming *E. coli* strains to medical devices and host tissues. Additionally, PNAG contributes to the evasion of host immune responses, thereby increasing the virulence of these strains. Elucidating the mechanisms underlying PNAG production and its role in biofilm formation is crucial for devising strategies to prevent and treat infections associated with biofilm-forming bacteria (Carzaniga et al., 2012; Cerca and Jefferson, 2008; Wang et al., 2023; Gholami et al., 2019).

Polysaccharide intercellular adhesin (PIA) is a crucial exopolysaccharide produced by *Staphylococcus epidermidis* (*S. epidermidis*), primarily responsible for biofilm formation, a key virulence factor in nosocomial infections. PIA enhances the bacterium’s ability to adhere to surfaces, including indwelling medical devices, and plays a significant role in evading the host immune response by inhibiting neutrophil phagocytosis and complement activation (Aarag Fredheim et al., 2011; Cheung and Otto, 2010; Fey and Olson, 2010; François et al., 2023; Mirzaei et al., 2019). Structurally, PIA is a linear polymer of *β*-1,6-linked *N*-acetylglucosamine, contributing to the biofilm matrix’s architecture and stability (Le et al., 2018; Sabaté Brescó et al., 2017; Mirzaei et al., 2016). Notably, PIA exhibits similarities to PNAG, which is found in other bacterial species such as *E. coli.* Both polysaccharides facilitate biofilm formation and immune evasion, although their specific mechanisms and structural features may differ. The presence of PNAG in *E. coli* also enhances its capacity to form biofilms and resist phagocytosis, highlighting a common evolutionary strategy among pathogenic bacteria to persist in hostile environments (Cheung and Otto, 2010; Rupp et al., 2001).

Secreted antibodies are vital in inhibiting bacterial biofilm production by targeting specific bacterial components and disrupting the biofilm matrix. These antibodies can bind to extracellular polysaccharides and proteins essential for biofilm integrity, thereby preventing bacteria from adhering to surfaces and each other (Lyu et al., 2020; Tvilum et al., 2023). Also, antibodies can form immune complexes with bacterial antigens, which may promote phagocytosis and clearance of biofilms by immune cells (Abdelhamid and Yousef, 2023). The effectiveness of these secreted antibodies highlights their potential as therapeutic agents in combating chronic infections associated with biofilms that are resistant to conventional antibiotic treatments (Zhao et al., 2023). In summary, the strategic utilization of secreted antibodies represents a promising approach to disrupting biofilm formation and enhancing bacterial clearance in clinical settings.

The polysaccharide PNAG in *E. coli* is similar to the PIA of *S. epidermidis*. This polysaccharide helps bacteria adhere to surfaces and each other during biofilm formation. This study aimed to investigate whether antibodies produced against PNAG/PIA can prevent the formation of biofilm structures. In this study, the effects of secreted antibodies against PNAG/PIA were evaluated for their inhibitory impact on biofilm formation in *Escherichia coli* ATCC 25922, a biofilm-forming strain, under *in vitro* conditions.

## 2 Materials and methods

### 2.1 Bacterial strains

Standard strains *S. epidermidis* 1,457, transmutant strain 1,457 (M-10/delta ica), and *E. coli* ATCC 25922 were provided by the Pasteur Institute of Iran.

### 2.2 Confirmation of strains

The colonies were confirmed using the standard microbial test outlined by the Clinical Laboratory Standards Institute 2023 (CLSI 2023) guidelines. In accordance with biochemical criteria, a susceptibility test was performed to confirm the transmutant strain 1,457 using novobiocin (20 μg), gentamicin (10 μg), ciprofloxacin (5 μg), and erythromycin (10 μg) (BD, BBL™ Sensi-Disc™) on Mueller Hinton agar (Difco, Becton–Dickinson, Franklin Lakes, NJ, United States; Mack et al., 1992).

### 2.3 *In vitro* biofilm formation assay

The biofilm formation assay was performed as previously described (McKenney et al., 1998). In summary, the optical density (OD600) of the inoculated colonies in BHI broth was adjusted to 0.7. Subsequently, 200 μL of the 1:200 diluted supernatants in BHI broth, enriched with 1% (w/w) D-glucose (BHIGlc) and 4% (w/w) NaCl (BHINaCl), was transferred to a polystyrene microtiter plate (Nunc, Roskilde, Denmark). After incubating for 24 h at 37°C, excess stain and planktonic cells were eliminated by washing each well three times with phosphate-buffered saline (PBS). Subsequently, each well received 150 μL of a 1% crystal violet solution. The stain bound to the adherent cells was subsequently dissolved in 160 μL of 30% acetic acid, and the absorbance was measured spectrophotometrically at 595 nm.

### 2.4 PIA extraction and purification method

The extraction and purification of PIA were performed according to established methods previously described in the literature (Mirzaei et al., 2016; Mack et al., 1994). In summary, bacterial strains cultivated in 2 liters of Tryptic Soy Broth (TSB) at 37°C with gentle agitation (40–50 rpm/min) for 24 h and then harvested by centrifugation at 1000 G for 20 min at 4°C. The cells were then resuspended in 20 mL of PBS (pH 7.5) and sonicated four times for 30 s each on ice. Following another centrifugation at 12000 rpm for 15 min at 4°C, the supernatant was collected using a Centriprep 10 device (Amicon, Witten, Germany). Soluble proteins were eliminated with proteinase K, and the sample was then transferred to a C18 column (KNAUER, Germany) pre-equilibrated with 50 mM PBS. The purified macromolecules were then stored at −20°C for preservation and future use (Mirzaei et al., 2016).

### 2.5 Confirmation of purified PIA by colorimetric assay

Suspected PIA fractions (100 μL) corresponding to the obtained peaks were evaluated using a colorimetric assay. This method facilitated the analysis of hexosamine in glycosaminoglycans under mild acid conditions (pH = 5). The interaction between 3-methyl-2-benzothiazolone hydrazone hydrochloride (MBTH) and the 2,5-anhydrohexoses, which result from the deamination of hexosamines, was assessed by the resulting color complex (Smith and Gilkerson, 1979).

### 2.6 Fourier-transform infrared spectroscopy

The infrared spectroscopic analysis of the purified polysaccharide was conducted utilizing the regularized deconvolution method (Buslov and Nikonenko, 1998). Powdered samples were transiently dispersed within KBr pellets, and the spectroscopic data was obtained utilizing a Bruker Vector 22 instrument, with an average of 256 scans on the FT-IR spectrometer (Buslov and Nikonenko, 1998).

### 2.7 Proton nuclear magnetic resonance spectroscopy (^1^H-NMR)

Previous studies have shown that recovered PIA can be analyzed using a ^1^H-NMR spectrum in deuterated water (D2O) (Haner and Keifer, 2007; Mack et al., 1999). For the experimental measurements, a sample with a concentration of 100 μg/mL of polysaccharide was utilized. The spectroscopic data was collected at a constant temperature of 25°C.

### 2.8 Immunization of mice

Female BALB/c inbred mice, aged 6–8 weeks (acquired from the Research Institution of Pasteur Karaj, IR Iran), were allocated into three groups of six. These mice were accommodated in standard stainless steel cages, maintained at 23–25°C with 60–70% humidity, and subjected to a 12-h light/dark cycle for 1 week before the experiment. They had unrestricted access to a standard diet and water. Each mouse in the designated group received subcutaneous immunizations on days 0, 14, and 28 with 100 μg of the respective lyophilized antigens in 1% alum solution in PBS, following a serial dilution procedure and assessment by colorimetric assays. Booster doses were administered at 2-week and 4-week intervals following the initial immunization. Blood samples (500 μL) were collected from the orbital sinus of the six mice in each group 2 weeks post-injection. To obtain serum, the blood was centrifuged at 5000 rpm for 5 min and stored at −20°C (Amini et al., 2016).

The experimental groups were defined as follows:

G-I: PBS (100 μg);
G-II: PIA (100 μg);
G-III: Reserve PIA (100 μg).

### 2.9 Enzyme-linked immunosorbent assay

Anti-PIA antibodies were introduced into the immunized mice’s sera using a commercial enzyme-linked immunosorbent assay following each immunization. In brief, 96-well plates (Extra gene, United States) were coated overnight with 100 μL of PIA (1 μg/well) in PBS at 4°C. Subsequently, the plates were washed three times with washing buffer [0.05% (v/v) Tween 20 in PBS] and blocked with PBS/Tween 20 containing 5% bovine serum albumin (BSA) for 2 h at 37°C. Following blocking and washing, the mouse sera diluted in blocking buffer were added to the wells in duplicate, 100 μL per well. The plates were then incubated for 2 h at 37°C, washed three times, and incubated with HRP-conjugated anti-mouse IgG (Sigma, United States) diluted 1:10000 as a secondary antibody at 37°C for 2 h. After washing, as previously mentioned, enzymatic activity was observed by adding 100 μL of tetramethylbenzidine (TMB) substrate. The reaction was terminated after a duration of 30 min by adding 100 μL of 2 N H_2_SO_4_.

### 2.10 Opsonophagocytosis assay

**Figure 1:**
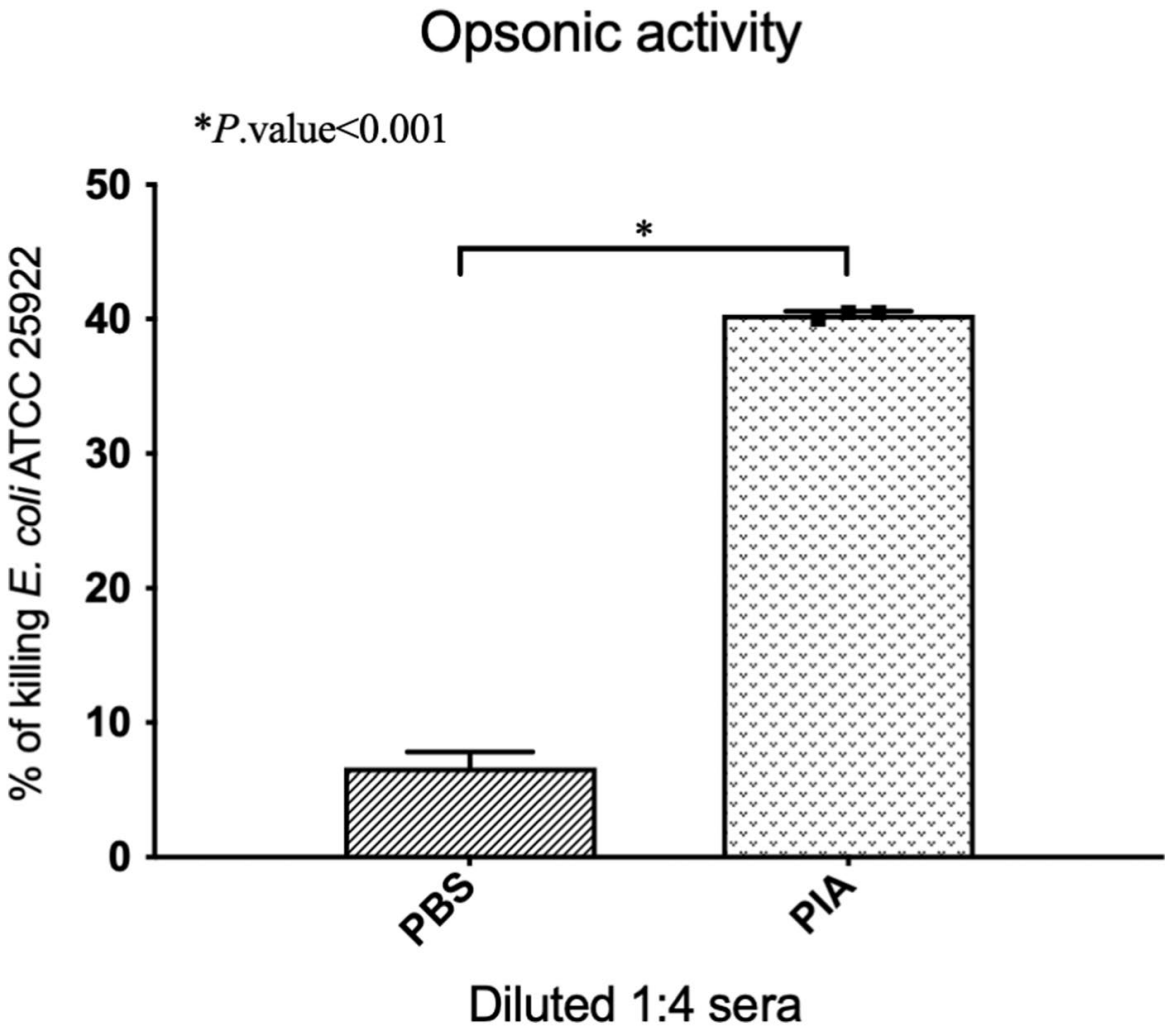
Comparative analysis of opsonophagocytic killing activity against *E. coli* ATCC 25922 strain by antibodies from mice immunized with antigens in various formulations. Serum-killing activity was measured by taking sera from groups of mice, followed by incubation with bacterial strains and mouse macrophages in the presence of rabbit complement. The data represent the mean ± sd of the percentage of bacteria phagocytized compared to PBS-treated samples. Based on the results, pooled sera from PIA group mice at a dilution of 1:4 were able to kill about 40.48% of bacteria in the presence of mouse macrophages compared to normal serum.

Based on the methods previously described, we assessed the susceptibility to opsonic killing (Farjah et al., 2015; Ames et al., 1985; Figure 1). In these experiments, *E. coli* ATCC 25922, a native biofilm producer, was utilized. To summarize, the *E. coli* ATCC 25922 strain, cultured overnight in TSB, was collected using centrifugation (12,000 rpm for 5 min). It was subsequently washed twice with PBS and diluted to a concentration of 5 × 10^5^ CFU/mL. The assay involved mixing 100 μL of heat-inactivated serum samples (56°C for 30 min in a water bath) diluted 1:2 with 100 μL of the bacterial suspension and incubating at 37°C for 30 min. Following a PBS wash, 100 μL of mouse macrophages (10^6^ cells/ml) and 100 μL of 10% fresh infant rabbit serum, serving as a complement source, were added and incubated at 37°C for 90 min. Control tubes, which excluded antibodies, macrophages, or complement and used RPMI medium instead (Capricorn Scientific, Ebsdorfergrund, Germany), were included in each assay. Finally, a 100 μL sample was collected, diluted in PBS, and plated to assess bacterial counts (Farjah et al., 2015; Ames et al., 1985).

The opsonophagocytic killing assay was conducted in triplicate on distinct days.

The opsonic-killing activity of the serum is determined using the following calculation:


$$\begin{array}{l} {}[1-(c.f.u.\mathrm{immune serum}\;\mathrm{at}90\;\mathrm{minc}.f.u^{.-1}\mathrm{of}\;pre-\\ \mathrm{immune serum}\;\mathrm{at}90\;\min)]\times 100 \end{array}$$

Mouse macrophages, harvested intraperitoneally and cultured in RPMI medium, were utilized in the procedure above, which followed the injection and retrieval of peritoneal cavity fluid (Ray and Dittel, 2010). The viability of the cells was assessed utilizing the trypan blue exclusion assay. This technique was also employed to assess both the quantity and viability of macrophage cells, with duplicate measurements performed to ensure accuracy (Hu et al., 2005).

### 2.11 *In vitro* biofilm inhibition assay

**Figure 2:**
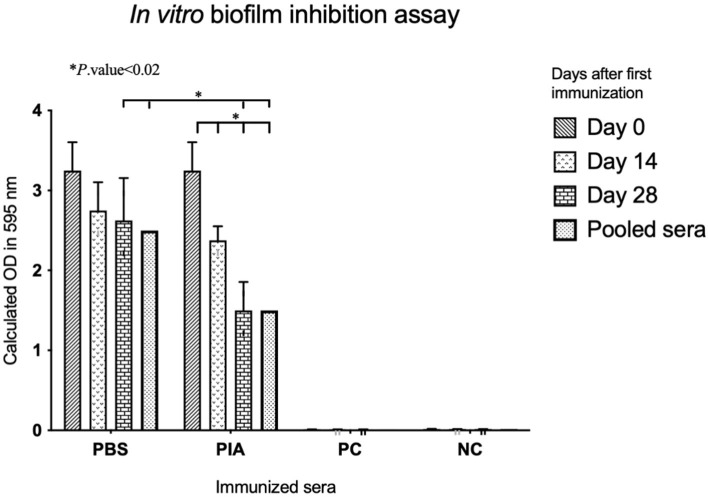
Comparative analysis of biofilm inhibitory effects of the sera. The biofilm-inhibitory effects of increasing antibodies (after each immunization) were evaluated by comparing the positive and negative controls. The downward trend in biofilm formation inhibition in terms of OD is evident in the graph, and the results were statistically significant.

Utilizing a semi-quantitative microtiter plate method, the study examined the impact of pre- and post-immune IgGs against injected antigens on the *in vitro* biofilm formation of *E. coli* ATCC 25922 (Shahrooei et al., 2012; Figure 2). Sterile TSB-glucose served as a negative control within the assay and was independently replicated three times (McKenney et al., 1998; Shahrooei et al., 2009). The *in vitro* biofilm formation assay had been previously detailed and published (Shahrooei et al., 2012). For each isolate, three replicate wells were seeded to ensure reproducibility and accuracy in the experiment.

### 2.12 Statistical analysis

A two-way multiple-group analysis of variance (ANOVA) was performed using GraphPad software to statistically analyze the results statistically, considering a *p*-value of less than 0.05 as significant.

## 3 Results

### 3.1 Confirmation of strains

Based on biochemical methods such as hemolytic pattern, Gram stain, and antibiotic susceptibility test by utilizing novobiocin disk, the provided colonies were confirmed to be *S. epidermidis*.

Although *S. epidermidis* was intrinsically sensitive to erythromycin, the transmutant strain 1,457 exhibited induced resistance to the specified antibiotic as a result of the *erm* gene in Tn917 (Mack et al., 1992).

Experimental data indicates that the wild-type strain, owing to the absence of abnormalities in the ica genes and the presence of PIA synthesis mechanisms during the accumulation phase, is capable of forming biofilm in TSB medium. On the contrary, transmutant strain 1,457, through the destruction of the current structure of the responsible genes for PIA synthesis, cannot produce biofilm in the mentioned mediums.

The level of crystal violet content in the wild specimen wells showed that this strain could produce biofilm in different conditions. In contrast, the mutant strain has lost the biofilm formation ability because of a mutation in the genes responsible for PIA synthesis.

### 3.2 Characterization of the PIA

**Figure 3:**
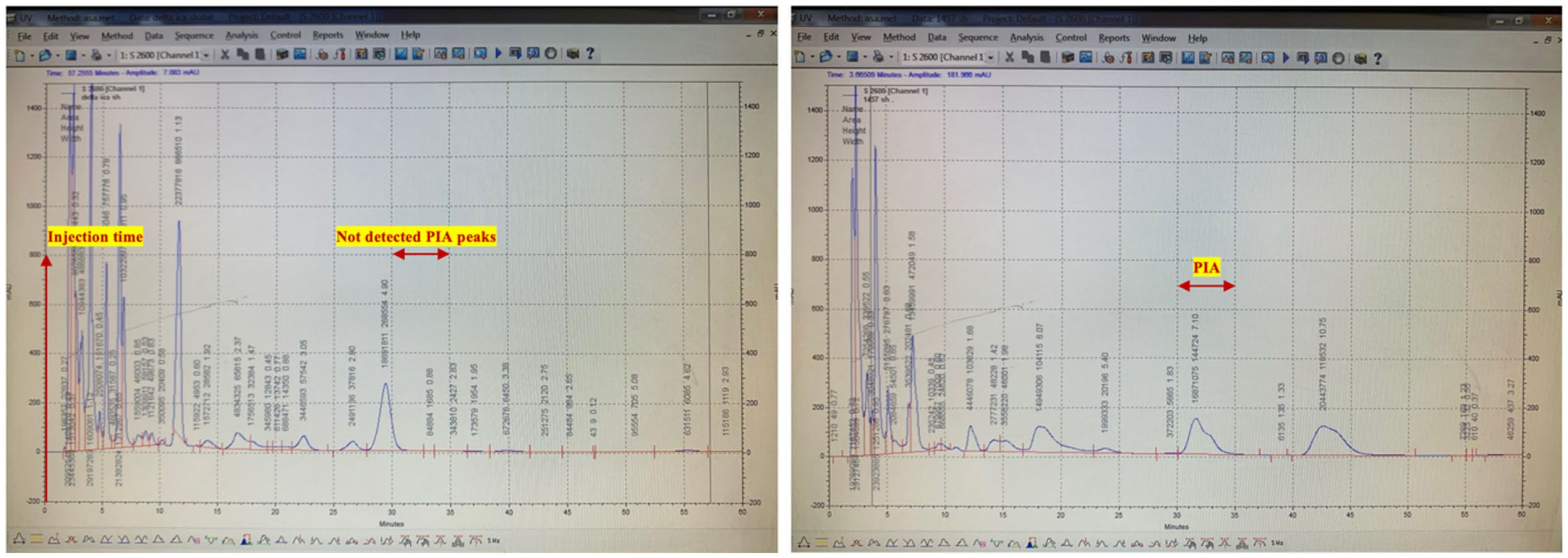
Purification of PIA by size exclusion chromatography (SEC). Representative HPLC chromatogram for native PIA. The sample in a final volume of 1 mL was introduced after 1 h of column equilibration. PIA was eluted near the void volume 30 min after sample injection by using a 1 mL/min flow rate. Polysaccharide was detected at a wavelength of 206 nm.

Parallel analyses of both strains by HPLC indicated that our aforementioned polysaccharide would be eluted near the void volume (100 KDa in branched forms) of the column in the *S. epidermidis* 1,457 types extract, still transmutant strain 1,457 did not produce detectable amounts of a specific polysaccharide antigen, conversely, the spontaneous variants produced significantly lower quantities of antigen. The chromatograms of wild (1457) and the HPLC analysis of the transmutant strain 1,457 is illustrated in Figure 3.

The polysaccharide fractions isolated from the HPLC chromatograms were evaluated using a colorimetric assay, with hexosamine used as the standard. In this assay, hexosamine acted as a positive control, and a standard curve was constructed using different concentrations of hexosamine. The cumulative peak concentration of PIA was then determined based on the hexosamine standard curve, revealing a concentration of 5,700 μg/mL for PIA and 10 μg/mL for transmutant strain.

**Figure 4:**
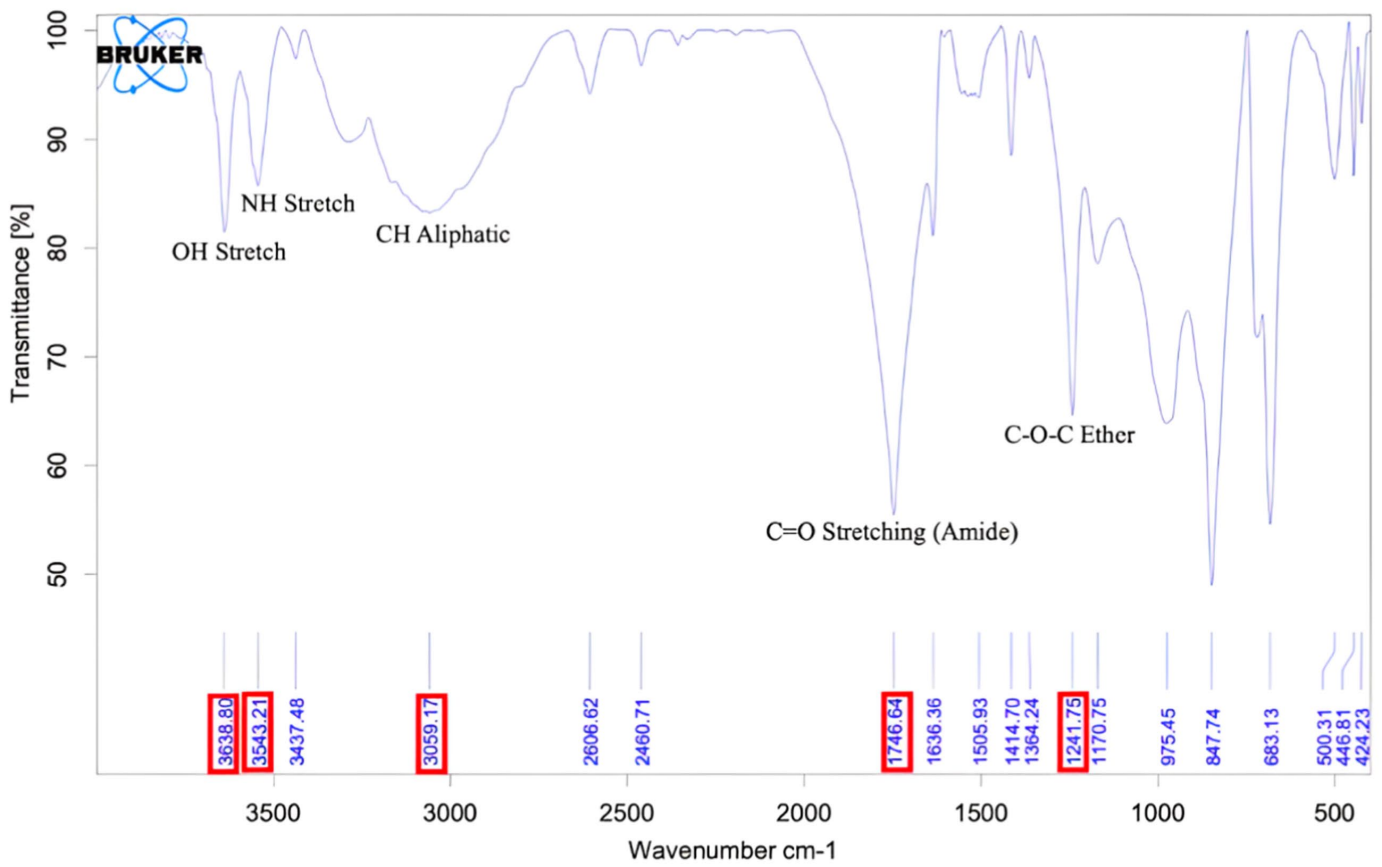
FT-IR graph of purified PIA. The peak at wavelength 1746 indicates the polysaccharide structure of the extracted compound, and the peak at wavelength 3,543 corresponds to the NH unit in PIA.

Figure 4 shows the IR spectra of PIA in the 4000–500 cm^-1^ 261 range and the result of this deconvolution.

**Figure 5:**
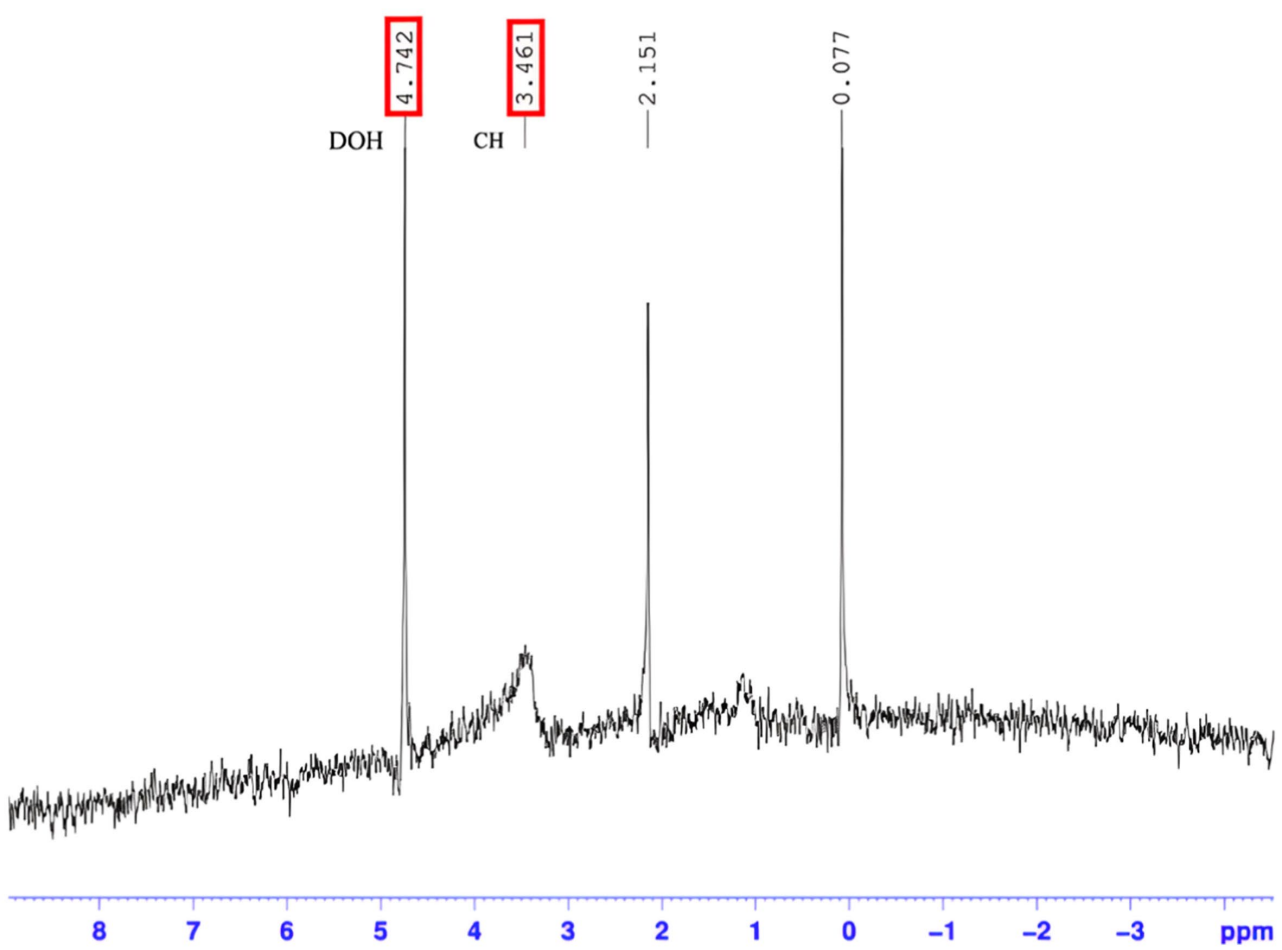
Resonance pattern for hydrogen atoms of purified PIA. The ^1^H NMR spectrum of the PIA polysaccharide confirms the presence of *N*-acetylglucosamine hydrogens between peaks 0.2–5. The 0.2 ppm peak is related to NH hydrogens, the 0.5 ppm peak is related to alkane hydrogens, the 3 ppm peak is related to aromatic hydroxyls of *N*-glucosamine, and the 4–5.

Detailed data of the ^1^H-NMR spectra of the recovered PIA is illustrated in Figure 5.

### 3.3 Anti-PIA humoral response

**Figure 6:**
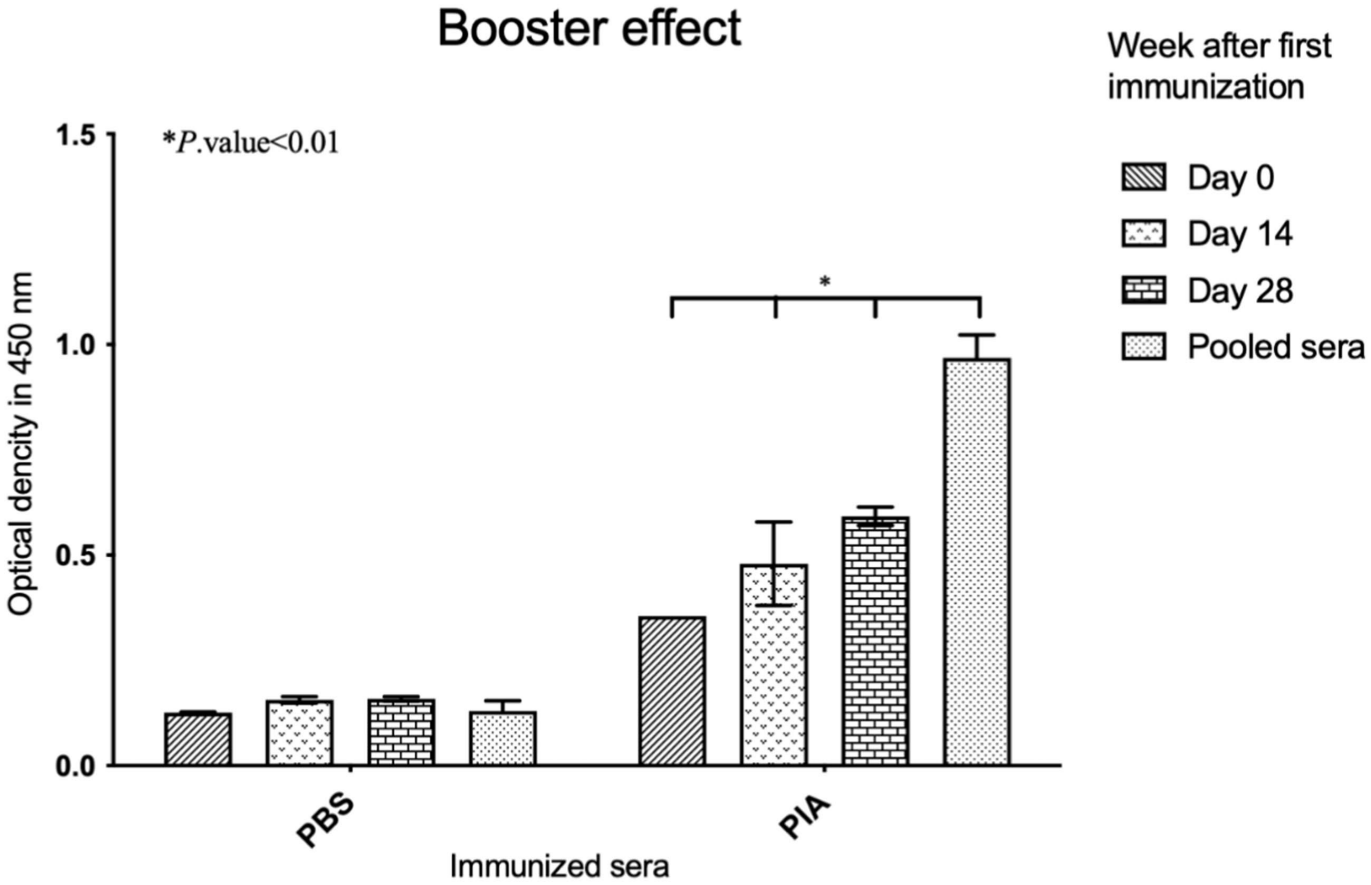
Evaluation of induced IgGs in immunized mice. ELISA was conducted by coating the native PIA, and the increase in antibodies was assessed for immunized sera compared to the controls. Significant effects appeared following the administration of the booster. Six weeks after the first injection, the titers of antibodies had increased. Antibody titration was assessed as 1: 100.

Mice serum antibody titers were examined (≤1: 100) using antigen-mediated ELISA to evaluate the total IgG antibody response against PIA. The results indicated that significant differences were observed when comparing the PBS and PIA groups. While the PBS group showed relatively little change over time (with a small and significant increase from day 0 to day 14 and 28), the PIA group exhibited a significant and sustained increase in values. It is worth noting that the PIA-immunized groups had significantly higher and higher titers of IgG antibodies than the control group (PBS) at all time points. In mice vaccinated with PIA and after 14 days, very little IgG against PIA was produced (*p*- value<0.164). With the injection of the first booster dose, a change in antibody titer was observed (*p*- value<0.003). After the injection of the second booster dose (day 42), a change in the IgG trend was observed (*p*- value<0.003). The results were evaluated by comparing the groups that received PIA at different time points (Figure 6).

### 3.4 Opsonophagocytic killing activity

The functional activity of raising antibodies against PIA in the presence of collected mouse macrophages and infant rabbit complement was determined using an opsonophagocytosis assay. To evaluate the phagocytosis ability of *E. coli* ATCC 25922 by anti-PIA IgG, the bacteria were incubated with a 1:4 dilution of anti-PIA antibody (serum), mouse macrophages, and rabbit complement. The tests were conducted in duplicate, and the results are shown in Figure 1. According to the results, the pooled sera of mice from the PIA group at 1:4 dilution were able to kill about 40.48% of the bacteria in the presence of mouse macrophages compared to normal serum (Figure 1).

### 3.5 *In vitro* biofilm inhibition assay

To assess the biofilm inhibitory effect, pre-immune and post-immune sera were tested by a semi-quantitative biofilm inhibition procedure. Experiments were conducted using sera from mice that were boosted at specific time intervals (14, 28, and 42 days), and results were obtained by comparing sera from immunized and non-immunized subjects, respectively. Following the investigation of the effect of antibodies on the biofilm formation process, the results indicated that sera immunized with pure polysaccharide showed a significant difference compared to non-immunized serum (*p*-value = 0.001). Also, in the PIA group, significant differences were observed between day 0 and day 14 (*p*- value =0.010), day 0 and day 28 (*p*- value =0.005), and day 0 and pooled serum (*p*-value <0.001). These findings indicated that the effect of PIA intervention increases over time. The difference between day 14 and day 28 was insignificant (*p*- value =0.074), but the comparison of day 14 with pooled sera showed a significant difference (*p*- value =0.010). These results indicated that the effectiveness of PIA intervention increased continuously from day 0 to day 28 and reached its peak in pooled serum samples (Figure 2).

## 4 Discussion

*Escherichia coli* is a major cause of gastrointestinal diseases, urinary tract infections (UTIs), neonatal meningitis, and sepsis. However, the potential for vaccination against these strains has been less evaluated due to the chemical and serological diversity of effective target antigens, namely lipopolysaccharides and capsular polysaccharides. Finding a conserved surface polysaccharide among various *E. coli* strains could increase the potential for using such an antigen as a vaccine. Furthermore, if this antigen is expressed by other phylogenetically diverse but common pathogens, such as *staphylococci*, the interest in its vaccine potential is heightened.

As a first step in determining whether PIA/PNAG has broad vaccine potential, we examined recent findings by Wang *et al.*, which showed that *E. coli* strains with sequenced genomes all possess a genetic locus homologous to the *staphylococcal icaADBC* locus, which they named *pgaABCD* (Wang et al., 2004). The presence of the *pga* locus is associated with PNAG production. In both *Staphylococcus* and *E. coli*, the PIA/PNAG polymer causes biofilm formation and is related to the cell surface. In *staphylococci*, PIA is identified as a virulence factor and a target for protective antibodies. *E. coli* isolate ATCC 25922 contains the *pga* genetic locus homologous to *ica* and produces PNAG that immunologically cross-reacts with PNAG isolated from *Staphylococcus aureus* (*S. aureus*). PNAG production by *E. coli* allows active opsonic antibodies produced against the PNAG antigen of *S. aureus* to eliminate these organisms *in vitro* culture (Nguyen et al., 2020; Wu et al., 2024; Peng et al., 2023; De Vor et al., 2020; Itoh et al., 2008; Dong et al., 2019).

Biochemical and molecular methods are helpful for the accurate identification of bacteria. In this study, the strains utilized in the initial stage were assessed through biochemical processes, and all strains were confirmed to be *S. epidermidis*.

The preparation of isogenic biofilm and PIA-negative strain based on the phage mutagenesis method showed that *Tn917* is directly integrated as a sequence at a specific site (*ica A*), and this operation leads to insertion mutation in the *icaADBC* operon in strain 1,457. After performing the phage mutagenesis method, PIA and biofilm-negative strain (isogenic *S. epidermidis* 1,457 strain) eventually appeared.

Two PIA-positive and negative strains (*S. epidermidis* 1,457 and transmutant strain 1,457) were grown in parallel under identical conditions, and the target polysaccharide was purified. Our results confirm previously published findings (Maira-Litrán et al., 2005; Davison and Baddiley, 1963).

Gel chromatography was an effective method for purifying macromolecules (Spiliopoulou et al., 2012). PIA was isolated at a flow rate of 1 mL/min, and 5 mL fractions were collected every 5 min using a fraction collector. Representative PIA fractions were assessed using colorimetric analysis (Mack et al., 1994; Spiliopoulou et al., 2012).

PIA weighs approximately 100 kDa and is soluble in buffers, according to findings by Maira-Litrán et al. (2002). Given the effect of removing impurities from the antigen on increasing functional antibodies in this study, impurities were removed by additional centrifugation (Maira-Litrán et al., 2005; Spiliopoulou et al., 2012).

Pure PIA was eluted within 30 min after injection. After colorimetric tests, pure PIA contains significant amounts of hexosamine, reaffirming findings from previous studies (Mack et al., 1996).

FT-IR has been successfully used to analyze the composition and structure of complex chemical compounds (Ziebuhr et al., 1999). By employing the deconvolution method, the spectral characteristics of polysaccharides with glycosidic bonds can be defined within the range of 4,000–500 cm^−1^. According to FT-IR patterns, regions where stretching vibrations *ν*(CO) C–O–C of the glycosidic bridge in oligosaccharides appear are present in the spectra of the 1,175–1,140 cm^−1^ region, which is close to the region mentioned in previous work. In the spectral analysis, the C=O stretching band was detected at 1748 (PIA) cm^−1^ (Kacuráková and Mathlouthi, 1996).

^1^HNMR spectroscopy has been employed as a precise method for characterizing polysaccharides extracted from *Staphylococcus,* as previously mentioned in (Zielinski et al., 1991). In this research, one-dimensional ^1^H-NMR spectra were captured using the normal acquisition mode. The NMR spectrum of PIA shows signals attributed to *N*-acetyl-*D*-glucosamine (3R,4R,5S)-3-amino-6-(hydroxymethyl)oxane-2,4,5-triol) residues linked to b-1,6 at 3 ppm. Comparable data have been obtained in previously published studies (Kacuráková and Mathlouthi, 1996; Smith and Gilkerson, 1979). Mack reported that the H-2 proton of *N*-acetylated *D*-glucosamine residues exists at a resonance of 2.9 ppm. In this study, the correlation signal for the mentioned residues was also detected as a narrow peak at 2.8 ppm (Kacuráková and Mathlouthi, 1996).

Research on the attachment of *S. epidermidis* to biomaterials has mainly focused on the role of extracellular polysaccharides (Ziebuhr et al., 1999). Simultaneous analysis of PIA-positive and PIA-negative biofilms was conducted using 96-well microtiter plates. The resulting biofilm formation showed that wild-type *S. epidermidis* is a biofilm-forming strain, still transposon mutant strain could not produce biofilm due to disruption of the *ica* gene (Shahrooei et al., 2009). These results are consistent with previous study results. The results suggest that although multiple factors can influence biofilm formation in *S. epidermidis*, PIA formation by manipulating of the *ica* locus is significant in the aggregation stage (Hernández-Cuellar et al., 2023). Although PIA/PNAG is weakly immunogenic, biofilm inhibitory effects have been reported from antibodies produced against this antigen.

In this study, according to the deacetylation of the polysaccharide (PIA/PNAG) and the presence of amino groups, IgG titers increased after booster doses. ELISA results showed that day 14 sera did not show any inhibitory effect against *E. coli* biofilm formation. At the same time, following booster doses, significant inhibition appeared in sera tested at 28 and 42 days, compared to sera immunized at 14 days (*p*- value<0.003). The PIA antigen produced antibodies and also increased levels of protective IgG titers.

Although an increase in IgG antibody titer is considered a good criterion for enhancing immunity, what is more critical in defense and resistance against strains is the efficiency and quality of these antibodies in opsonizing and phagocytic killing of this bacterium. Therefore, to investigate the biological function of produced antibodies *in vitro*, an opsonophagocytosis test was conducted to evaluate the biological activity of antibodies in binding to the antigen, as well as the process of opsonization and elimination of bacteria by macrophage cells. Research demonstrated that administering PIA to laboratory animals can opsonize 40.48% of bacteria at a 1:4 serum dilution. This experiment indicates that anti-PIA antibodies can eliminate bacteria by attaching to them through phagocytic cells.

Following an investigation of the effect of antibodies on the biofilm formation process, the results indicated that sera immunized with pure polysaccharide showed a significant difference compared to non-immunized serum. Also, in the PIA group, significant differences were observed between day 0 and day 14, day 0 and day 28, and day 0 and pooled serum. These findings indicated that the effect of PIA intervention increases over time. The difference between day 14 and day 28 was insignificant, but the comparison of day 14 with pooled sera showed a considerable difference. These results indicated that the effectiveness of PIA intervention increased continuously from day 0 to day 28 and reached its peak in pooled serum samples.

Although further studies are needed to determine whether anti-PIA/PNAG antibodies can protect against *E. coli* infections, these initial studies confirm the potential of PIA/PNAG as a protective vaccine for diverse and common pathogens such as *E. coli*, *S. aureus*, and coagulase-negative *staphylococci*. Furthermore, if pathogens such as Bordetella species, *Yersinia* species, and others that have a homologous *pga* genetic locus also produce PIA/PNAG in a manner and form that antibodies are opsonic and protective to the antigen, then a properly constructed PIA/PNAG-based vaccine could protect against a wide range of human and animal pathogens (Cerca et al., 2007; Skurnik et al., 2016).

If the secreted anti-PIA antibody exhibits suboptimal performance, utilizing a protein construct in conjunction with the compound as a conjugate may enhance the efficacy of the anti-PIA antibodies, warranting further investigation.

## 5 Conclusion

This study investigated the effect of antibodies produced against PIA on the biofilm formation process in *E. coli*. The results indicated that PIA purified from *S. epidermidis* 1,457 was capable of inducing the production of protective antibodies with opsonizing properties against ATCC 25922, and also inhibited biofilm formation *in vitro* in *E. coli*. Also, the groups of mice receiving PIA showed a significant increase in antibody production and protective IgG titer levels compared to the control group.

These findings may open a new avenue for future research in controlling biofilm formation in *E. coli* and other related bacteria.

## Data Availability

The datasets presented in this study can be found in online repositories. The names of the repository/repositories and accession number(s) can be found in the article/supplementary material.
